# Eliminating Loneliness and Enhancing Social Support: Findings from Community Focus Groups

**DOI:** 10.1093/geroni/igaf122.2989

**Published:** 2025-12-31

**Authors:** Vivian Miller, Caleb Skinner, Daniel Maitland

**Affiliations:** Bowling Green State University, Bowling Green, Ohio, United States; Bowling Green State University, Bowling Green, Ohio, United States; University of Missouri-Kansas City, Kansas City, Missouri, United States

## Abstract

Social support and social connection are vital for older adults. Older adults may be at increased risk for loneliness and a lack of social support as this cohort may live alone, experience the loss of friends, or have chronic illness. Failure to have social connections and social support results in significant consequences. Consequences of a lack of connection and support include risk for mental illness, risk for cognitive decline, and risk for physical health concerns (e.g., high blood pressure). To mitigate the consequences of loneliness and to increase support efforts, it is important to explore the lived experiences of older adults with a focus on time periods, activities, and engagement that promoted a sense of support. A total of seventeen (N = 17) participants across two focus groups engaged in this study. Through semi-structured interviews, participants shared that they felt supported by participating in enriching experiences at their local senior center. Through transportation provided by their senior center, community members otherwise transportation disadvantaged were able to engage with others, establish a sense of belonging, and maintain support through programs and services. Additionally, multiple participants explained that their church and pastor provided a sense of connection and were instrumental to their support system after the loss of loved ones. Findings from this study indicate the need to promote senior centers as a community focus. Additionally, findings suggest that senior centers can be a resource through networking of members and attendees. Authors share policy, research, and program implications.

